# Four-Fold Multi-Modal X-ray Microscopy Measurements of a Cu(In,Ga)Se_2_ Solar Cell

**DOI:** 10.3390/ma14010228

**Published:** 2021-01-05

**Authors:** Christina Ossig, Christian Strelow, Jan Flügge, Andreas Kolditz, Jan Siebels, Jan Garrevoet, Kathryn Spiers, Martin Seyrich, Dennis Brückner, Niklas Pyrlik, Johannes Hagemann, Andreas Schropp, Romain Carron, Gerald Falkenberg, Alf Mews, Christian G. Schroer, Tobias Kipp, Michael E. Stuckelberger

**Affiliations:** 1 Deutsches Elektronen-Synchrotron (DESY), Notkestr. 85, 22607 Hamburg, Germany; christina.ossig@desy.de (C.O.); jan.garrevoet@desy.de (J.G.); kathryn.spiers@desy.de (K.S.); martin.seyrich@desy.de (M.S.); dennis.brueckner@desy.de (D.B.); niklas.pyrlik@desy.de (N.P.); johannes.hagemann@desy.de (J.H.); andreas.schropp@desy.de (A.S.); gerald.falkenberg@desy.de (G.F.); christian.schroer@desy.de (C.G.S.); 2 Fachbereich Physik, Universität Hamburg, Luruper Chaussee 149, 22761 Hamburg, Germany; 3 Fachbereich Chemie, Universität Hamburg, Grindelallee 117, 20146 Hamburg, Germany; strelow@chemie.uni-hamburg.de (C.S.); jan.fluegge@chemie.uni-hamburg.de (J.F.); andreas.kolditz@chemie.uni-hamburg.de (A.K.); jan.siebels@chemie.uni-hamburg.de (J.S.); mews@chemie.uni-hamburg.de (A.M.); kipp@chemie.uni-hamburg.de (T.K.); 4 Empa, Überlandstrasse 129, 8600 Dübendorf, Switzerland; romain.carron@empa.ch

**Keywords:** CIGS, X-ray imaging, XBIC, XEOL, XRF, ptychography, scanning microscopy, multi-modal X-ray microscopy

## Abstract

Inhomogeneities and defects often limit the overall performance of thin-film solar cells. Therefore, sophisticated microscopy approaches are sought to characterize performance and defects at the nanoscale. Here, we demonstrate, for the first time, the simultaneous assessment of composition, structure, and performance in four-fold multi-modality. Using scanning X-ray microscopy of a Cu(In,Ga)Se${}_{2}$ (CIGS) solar cell, we measured the elemental distribution of the key absorber elements, the electrical and optical response, and the phase shift of the coherent X-rays with nanoscale resolution. We found structural features in the absorber layer—interpreted as voids—that correlate with poor electrical performance and point towards defects that limit the overall solar cell efficiency.

## 1. Introduction

In our ever growing demand for sustainable energy, thin-film solar cells offer a low-cost solution for green energy generation [1,2]. As solar cells are only as good as their weakest part, the efficiency of solar cells with polycrystalline absorber layers, such as Cu(In,Ga)Se${}_{2}$ (CIGS), is often limited by nanoscale defects and inhomogeneities, e.g., at grain boundaries [3,4]. This motivates the characterization of composition and performance at the nanoscale, but traditional laboratory-based techniques are challenged by the requirements of being non-destructive for operando measurements, while providing nanoscale spatial resolution and sensitivity to the buried absorber layer. This three-fold tradeoff cannot easily be satisfied: for example, the spatial resolution in electron beam-induced current measurements is linked to the surface sensitivity, which raises the need to alter the device by removing the upper layers [5] of the cell for 2D mapping of the solar cell absorber. Or, secondary ion mass spectrometry gives access to the depth profile of elemental distributions [6], but the investigated sample volume is destroyed by the measurement procedure. On the other hand, non-destructive methods with high penetration depth, like laser beam-induced current, photoluminescence, or X-ray fluorescence (XRF) measurements with an X-ray tube, are limited by their spatial resolution [7,8,9]. In contrast, hard X-ray synchrotron radiation is ideal to study these layered systems, meeting all three requirements simultaneously: due to its high penetration depth, buried structures in fully assembled devices can be characterized with unparalleled sensitivity and spatial resolution in a non-destructive way [10,11].

So far, scanning hard X-ray microscopy of solar cells has been used predominantly for the correlation of the elemental distribution with the electrical performance [12,13,14]. Only recently, measurements of the intra-grain strain have been included [15,16,17].

For an overall picture of the solar cell, including the complex interplay between synthesis conditions and performance, the simultaneous evaluation of different modalities is desirable. Therefore, we demonstrate, in this work, the further expansion of measurement modalities, combining the more established measurements of X-ray fluorescence (XRF) and X-ray beam-induced current (XBIC) with X-ray excited optical luminescence (XEOL) and ptychography. All these four methods are shortly introduced in the following.

*X-ray fluorescence (XRF):* When an inner-shell electron is excited by an incident X-ray photon, it leaves a core hole behind. Upon filling the core hole by an outer-shell electron, either an Auger transition occurs, or an X-ray photon is released with the energy corresponding to the energy difference of the electron states that is specific to the element. This latter process is denoted XRF, and the evaluation of the energy of the emitted X-ray photon leads to the element identification in the specimen [10,18,19].

*X-ray beam-induced current (XBIC):* The absorption of an X-ray photon in the absorber layer of the solar cell leads to a particle shower of electrons and photons and, after thermalization, to multiple excited electron-hole pairs at the band edges (simulations of this process are detailed in the supplementary information (SI) of Reference [20]). Same as upon excitation by photons in the visible range, the electrons and holes can be separated within the solar cell and collected at the solar-cell contacts. The resulting current is known as XBIC that is highly sensitive to recombination losses [13,21,22]. We have recently achieved an increase of the signal-to-noise ratio of XBIC measurements by orders of magnitude through the implementation of lock-in amplification [23].

*X-ray excited optical luminescence (XEOL):* In an analogous way to photoluminescence measurements with laser excitation, XEOL measurements assess the solar-cell performance with respect to radiative recombination of the X-ray excited electron-hole pairs by detection of the emitted photons with an energy corresponding to the bandgap of the solar-cell absorber [24,25]. Competing non-radiative recombination paths, e.g., through trap states at grain boundaries, may reduce the charge-carrier lifetime, thus decreasing the XEOL intensity.

*Ptychography:* Ptychography is used to iteratively reconstruct the phase shift of a coherent X-ray beam when passing through a material from the distribution of scattered photons measured with an area detector [26,27]. For the algorithm to converge, an overlap of the sampled beam spots is necessary. To describe the propagation of X-rays through a material, a complex refractive index,

(1) n=1−δ+iβ,

can be used, where δ describes phase shift, and β attenuation. The phase shift is correlated to the material’s electron density $\rho_{e}$ through

(2) $$\delta =\frac{\rho_{e}r_{e}\lambda^{2}}{2\pi },$$

where $r_{e}$ is the classical electron radius, and λ is the X-ray wavelength [28,29]. Compared to incoherent X-ray imaging, e.g., through absorption contrast, ptychography offers a greater spatial resolution that is rather limited by the signal captured under the largest scattering angle on the detector than by the spot size of the incident beam [30].

For the first time, we demonstrate here simultaneous raster-scan measurements with four-fold multi-modality combining XRF, XBIC, XEOL, and ptychography. We utilized this multi-modal measurement approach to correlate the composition with the electrical and optical performance, as well as the electron density, of a Cu(In,Ga)Se${}_{2}$ solar cell point-by-point.

## 2. Materials and Methods

### 2.1. Sample

The Cu(In,Ga)Se${}_{2}$ solar cell was synthesized on a polyimide foil substrate, as described in detail in Reference [31]. The Mo back contact layer (0.5 μm) was deposited by sputtering. The Cu(In,Ga)Se${}_{2}$ absorber layer was deposited by a multistage co-evaporation process around 450 °C substrate temperature and was subjected to an in-situ NaF+RbF post-deposition treatment in Se ambient. The integrated Ga:In composition ratio is 0.41:0.59, and the Cu(In,Ga)Se${}_{2}$ thickness is 3.16 μm. The CdS buffer layer (25 nm) was deposited in a chemical bath, followed by deposition of ZnO (65 nm) and transparent conductive oxide ZnO:Al (120 nm) layers by sputtering. The cell was contacted through an electron-beam evaporated Ni-Al grid and manually isolated by peeling-off surrounding Cu(In,Ga)Se${}_{2}$ material. Manually-applied silver paint (far from the region of interest) was used to electrically connect the Ni-Al grid to thin Cu wires and a dedicated printed-circuit board, from where co-axial wires were used for the connection to the current measurement circuit, as detailed in Reference [23].

**Figure 1:**
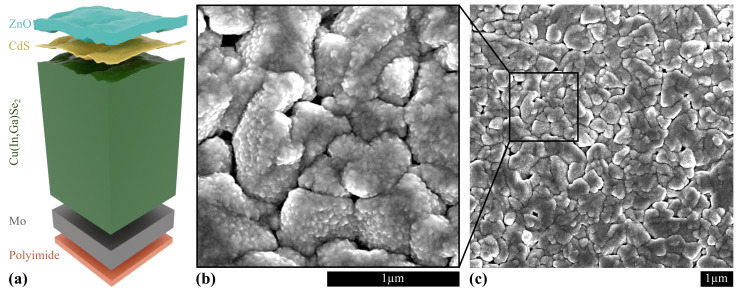
(**a**) Scheme of the Cu(In,Ga)Se${}_{2}$ (CIGS) solar-cell stack. (**b**,**c**) Scanning electron microscopy image of the solar-cell surface (courtesy of A. Jeromin, DESY NanoLab [32]).

Figure 1 shows a scheme of the Cu(In,Ga)Se${}_{2}$ sample, as well as two representative scanning electron microscopy (SEM) images of the cell surface (with ZnO:Al as uppermost layer), showing the granular structure of the absorber layer propagating to the solar-cell surface.

### 2.2. X-ray Microscopy Setup

**Figure 2:**
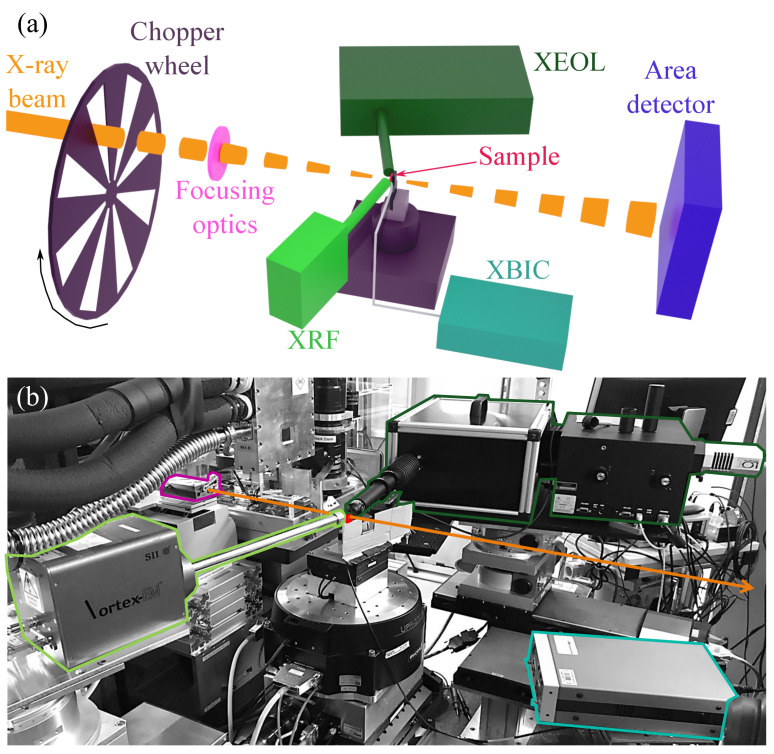
Experimental setup. (**a**) Scheme of the multi-modal X-ray microscopy measurement involving X-ray fluorescence (XRF), X-ray beam-induced current (XBIC), X-ray excited optical luminescence (XEOL), and ptychography. (**b**) Picture of the actual setup. The area detector for ptychography is located further downstream along the beam path and not visible here.

The experiment was performed in the microprobe hutch of the scanning X-ray imaging beamline P06 [33] of the synchrotron radiation source PETRA III, as laid out in Figure 2. Under the constraints of (i) limited space in the experimental hutch, (ii) limited solid angle for the X-ray optics and detectors, and (iii) minimized shielding by detector elements and the sample, the geometry displayed in Figure 2 was found to yield the optimum signal-to-noise ratio for all measurement modalities.

**Figure 3:**
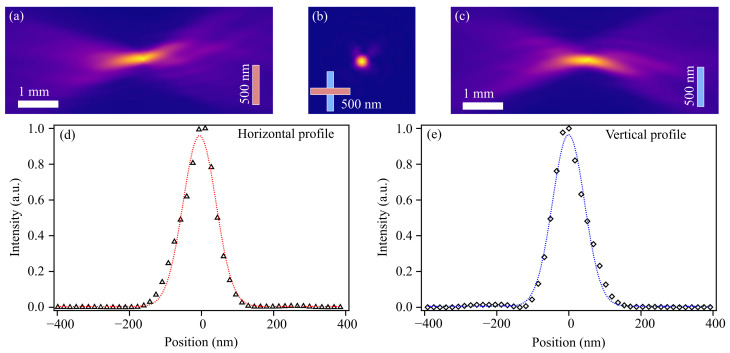
Characterization of the nano-focused X-ray beam by ptychography. (**a**) Beam caustic in the horizontal plane. (**b**) Beam-intensity distribution in the focus plane. (**c**) Beam caustic in the vertical plane. Line profiles through the focus for horizontal ((**d**), points) and vertical ((**e**), points) plane. The dotted lines are Gaussian fits that yield a FWHM of 108 nm for the horizontal plane and 105 nm for the vertical plane.

The photon energy was 15.25 keV, just above the Rb${}_{K}$ absorption edge (15.21 keV). Compound refractive lenses (CRL) made of Be, a corrective phase plate [34], and a pinhole were used to focus the beam. Figure 3 shows the caustic of the nano-focused X-ray beam as measured by ptychography. The coherent focus size was determined to 108nm(h)×105nm(v) in horizontal (h) and vertical (v) direction (FWHM), respectively. The photon flux was $7\times 10^{9}$ ph/s, resulting in a dose rate of $1.4\times 10^{13}$ eV/s in the solar-cell absorber.

*XRF:* For fluorescence measurements, a silicon drift detector (SII Vortex EM, Hitachi High-Tech Science Corporation, Tokyo, Japan) was used with a digital pulse processor (Xspress3, Quantum Detectors, Harwell Oxford, UK). The detector was placed in the plane of the storage ring inboard with an Al collimator; the angle between the sample surface and the detector was 7°, and the distance was 2 cm.

*XBIC:* For lock-in amplified XBIC measurements, the X-ray beam was modulated with a frequency of 8.015 kHz upstream of the X-ray optics as detailed in Reference [23]. After pre-amplification (SR570, Stanford Research Systems, Sunnyvale CA, USA) with 1 μA/V, the XBIC signal was demodulated by a lock-in amplifier (UHFLI, Zurich Instruments, Zurich, Switzerland) with a low-pass cutoff frequency of 501.1 Hz (8th order). The front contact of the solar cell was grounded to avoid artifacts from space charge at the cell surface and replacement currents that can be induced by the X-ray beam [13].

*XEOL:* The XEOL setup was placed in the plane of the storage ring outboard. The angle between the sample surface and the optical axis of the XEOL setup was 10°. The XEOL photons were collected with a 4× objective (UPlanSApo, Olympus, Tokyo, Japan) with a numerical aperture of 0.16, and the intensity was measured by a Si CCD camera (Andor iDus420 OE, Oxford Instruments, Abingdon, UK).

*Ptychography:* An area detector (Eiger X 4M, Dectris, Baden-Daettwil, Switzerland) was placed 8.05 m downstream of the sample, in the experimental nanoprobe hutch of beamline P06 [35], to measure the far-field diffraction patterns. The detector was set up in vacuum ($<10^{-3}\mathrm{mbar}$) with a flight tube to minimize the air path between sample and detector [36].

### 2.3. Measurement

For independent XRF, XBIC, XEOL, and ptychography measurements, different sets of measurement parameters would be ideal. Hence, the simultaneous measurement of all modalities requires a compromise between (i) low dose to limit sample degradation, (ii) high dose to maximize signal-to-noise ratio, (iii) high coherence to enable ptychographic reconstruction, and (iv) short dwell time to enhance throughput.

Balanced for the four measurement modalities, we performed a step scan where the fast shutter of the beamline was closed during position-settling time. We mapped an area of 4 μm×4 μm with 100 nm×100 nm step size at a nominal dwell time of 0.5 s per point. The actual dwell time was halved as the chopper fully blocked the beam 50% of the time. The effect of the chopper on the ptychographic reconstruction was deemed marginal in preliminary scans. The sample surface was perpendicular to the incident X-ray beam, and the scan motion was executed in the sample-surface plane.

Compared to dedicated scans optimized for each modality separately, the multi-modal measurement with the parameters specified above led to the following compromise:

*XRF:* The chopper required for high-sensitivity XBIC measurements blocks 50% of the photons, thus decreasing the statistics, which could easily be compensated by doubling the dwell time. In that case, there are no disadvantages of the multi-modal measurement approach compared to XRF-optimized measurements except for the lower throughput. For the absolute elemental quantification of the fluorescence data, we used PyMca 5.5.3 [37], where the extraction of mass fractions is based on the fundamental parameter method [38,39]; the background subtraction was done manually as motivated in Reference [40]. The self-consistent fits with self-absorption correction converged and are in excellent agreement with the average elemental distribution expected from the synthesis conditions. For further details on the quantification of the experimental results, we refer to the SI.

*XBIC:* For XBIC measurements, requirements are relaxed as long as a chopper is used to modulate the X-ray beam. Dedicated XBIC scans could be performed more than an order of magnitude faster with comparable signal-to-noise; the long dwell time required for the multi-modal measurement would only be a drawback if X-ray beam-induced sample damage was present, as is the case, e.g., in perovskite solar cells [20,41].

*XEOL:* As the most photon-hungry technique used in this experiment, the XEOL measurement determined essentially the minimum dwell time of the multi-modal measurement for satisfying signal-to-noise ratio. Accordingly, the penalty in signal quality by the 50% flux reduction by the chopper is highest in the XEOL signal. In addition, stray light from a multitude of electronic devices present in the X-ray hutch and mechanical vibrations compromised the signal-to-noise ratio.

*Ptychography:* The high-flux requirement for XRF and XEOL measurements required opening the beam-defining slits in the P06 optics hutch. While this allowed high signal-to-noise XRF and XEOL measurements, it comes at the cost of a reduced spatial coherence of the beam at the location of the X-ray microscope. As a consequence, the image quality in ptychography is reduced in this high-flux operation mode. In the presented measurements, a typical reduction in spatial resolution of less than about a factor of 2 was observed as compared to ptychographic measurements in high-coherence mode, while the focused flux was 10-fold higher. This trade-off between high focused flux and high spatial coherence will be relaxed at fourth-generation storage rings, such as PETRA IV [42].

For image registration, the enhanced correlation coefficient image alignment algorithm [43] from the OpenCV library [44] was used.

## 3. Results and Discussion

**Figure 4:**
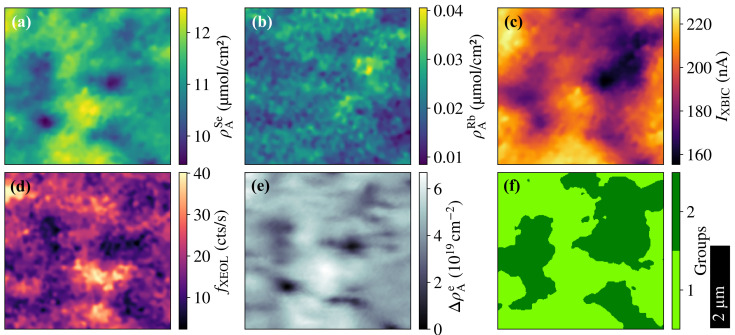
(**a**,**b**) Area density of Se ($\rho_{A}^{\mathrm{Se}}$) and of Rb ($\rho_{A}^{\mathrm{Rb}}$) from XRF measurements. (**c**) XBIC ($I_{\mathrm{XBIC}}$) from electrical performance measurements. (**d**) Photon count rate ($f_{\mathrm{XEOL}}$) from XEOL measurements. (**e**) Relative area density of electrons ($\Delta \rho_{A}^{e}$) from ptychography measurements. (**f**) Groups resulting from a *k*-means clustering of the maps shown in (**a**–**e**).

The maps showing the key results of the four modalities are displayed in Figure 4a–e. Resulting from XRF measurements, the area density of Se ($\rho_{A}^{\mathrm{Se}}$) and of Rb ($\rho_{A}^{\mathrm{Rb}}$) is shown in Figure 4a,b, respectively. The distribution of Se is representative of the main elements in the Cu(In,Ga)Se${}_{2}$ absorber layer that is followed also by the distribution of Zn in the ZnO layer (see Figure S1 for the distribution of all absorber elements and Zn). The distribution of Rb in the absorber layer is of particular interest due to the outstanding role of alkali elements for the Cu(In,Ga)Se${}_{2}$ solar-cell performance [45,46]. It is noteworthy that the relative variation of $\rho_{A}^{\mathrm{Rb}}$ is stronger compared to $\rho_{A}^{\mathrm{Se}}$, which can only partially be attributed to the smaller signal-to-noise ratio of the Rb quantification (note that $\rho_{A}^{\mathrm{Rb}}\ll \rho_{A}^{\mathrm{Se}}$); rather, this indicates a stronger Rb segregation.

The results from XBIC, XEOL, and ptychography measurements are shown in Figure 4c–e in terms of current ($I_{\mathrm{XBIC}}$), count rate of photons with an energy corresponding to the bandgap of the solar-cell absorber ($f_{\mathrm{XEOL}}$), and electron area density ($\Delta \rho_{A}^{e}$) relative to the minimum density of the mapped area (see Figure S2 for the conversion from phase shift to area electron density), respectively. Despite of the common measurement parameters being sub-optimal for each modality, Figure 4 demonstrates that a compromise can be found with high measurement quality in all four modalities.

Similar features, on a length scale from a few hundred nanometers up to one micrometer, are apparent in all maps of Figure 4a,c–e, while the features in the Rb distribution (Figure 4b) follow a different pattern resembling the inverted features of $I_{\mathrm{XBIC}}$ (Figure 4c). For correlative analysis of the four-fold multi-modal measurements, we followed two approaches.

**Figure 5:**
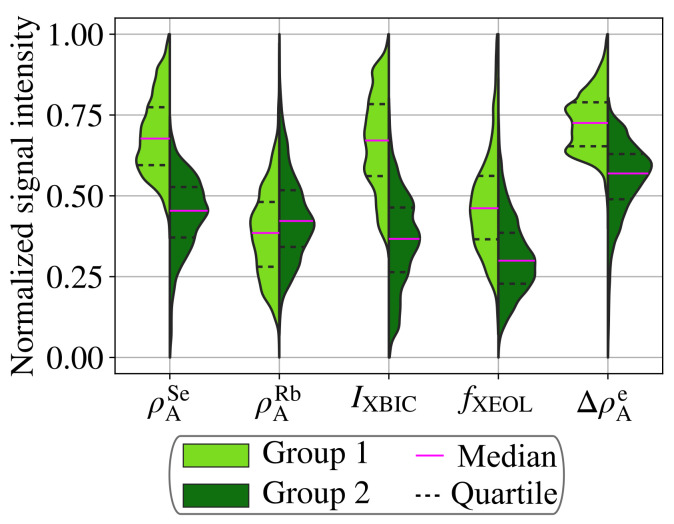
Violin plots of the 5 key measurands, split by the groups of the *k*-means clustering. The signal intensity is scaled such that 0 and 1 correspond to the minima and maxima of the maps shown in Figure 4 for the area density of Se ($\rho_{A}^{\mathrm{Se}}$) and of Rb ($\rho_{A}^{\mathrm{Rb}}$), of the XBIC ($I_{\mathrm{XBIC}}$), of the XEOL photon count rate ($f_{\mathrm{XEOL}}$), and of the relative electron area density ($\Delta \rho_{A}^{e}$). The distributions, scaled to the same area, are normalized such that their amplitude (shown on the horizontal axis) is proportional to the area with the corresponding measurement value for each group. Magenta lines indicate the median values, and dashed lines indicate the 25th and 75th percentiles.

First, we performed a *k*-means clustering analysis to classify those areas with common features in all modalities. Therefore, we applied tools from the python package scikit-learn [47] to the data sets from Figure 4a–e to group possible feature clusters. The *k*-means clustering algorithm uses an unsupervised machine-learning approach to sort data into a pre-selected number of groups, where the means of each feature at every point in a group are closer to the mean of their group than the means of any other group [48]. The optimal number of groups was identified to be two, based on the *elbow* and *silhouette* [49] methods as specified in the SI (see Figure S3). The result of the clustering is displayed in Figure 4f. Overall, group 1 predominantly includes areas of higher absorber area density and performance than group 2. The distribution of the measurement parameters is plotted separately for the two groups in Figure 5.

**Figure 6:**
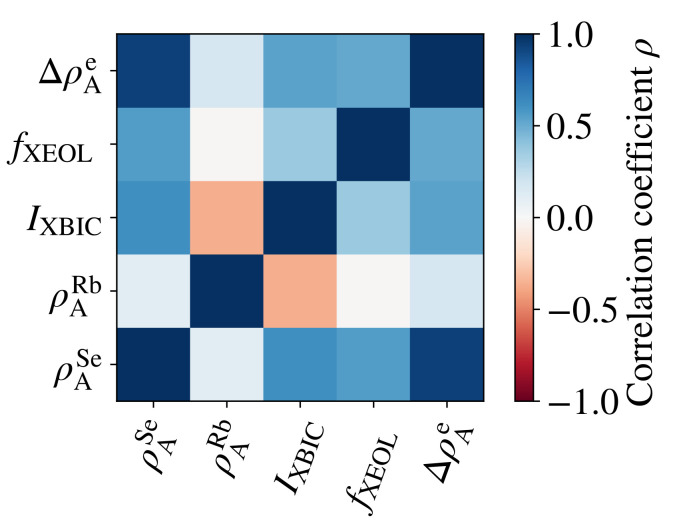
Correlation coefficient ρ for all combinations of the area density of Se ($\rho_{A}^{\mathrm{Se}}$) and of Rb ($\rho_{A}^{\mathrm{Rb}}$), of the XBIC ($I_{\mathrm{XBIC}}$), of the XEOL photon count rate ($f_{\mathrm{XEOL}}$), and of the relative electron area density ($\Delta \rho_{A}^{e}$).

Second, we evaluated the statistical correlation between the measurement parameters shown in Figure 4. The result is shown in Figure 6 as the matrix of the correlation coefficient ρ, where ρ=1, ρ=−1, and ρ=0 describe perfectly correlated, perfectly anti-correlated, and uncorrelated data, respectively [50]. For completeness, the statistical correlation within each group resulting from *k*-means clustering is provided in the SI (see Figure S4).

Considering the results from both the *k*-means clustering (Figure 5) and the correlative analysis (Figure 6) for the interpretation of the maps (Figure 4), we emphasize the following three observations:

*Topology:* When comparing the two raster-scanned maps in Figure 4a,e, the relative electron area density map shows higher resolution—resulting from the ptychographic reconstruction—than the fluorescence map, enabling us to see finer features in the topology of the absorber layer.

The similarity of features in the Se area density and relative electron area density maps of Figure 4a,e, the similarly shaped violins (Figure 5), and the positive correlation coefficient (Figure 6) of $\rho_{A}^{\mathrm{Se}}$ and $\rho_{A}^{e}$ are strong indications that both distributions have the same origin—the topology of the absorber layer. Thus, we can corroborate the claim made in Reference [51] that the ptychographically reconstructed phase shift and the absorber-element distribution represented here by Se are dominated by the absorber topology: the greater the number of atoms in the projected interaction volume of the beam with the absorber layer, the stronger are the fluorescence signal and the electron area density.

We presume two mechanisms to be responsible for the structures dominating the topology: first, the area density of the absorber layer tends to be smaller at grain boundaries than at grain cores [12,52]; second, voids and crevices in the absorber layer are known to be present in this type of solar cell [53], leading to low $\rho_{A}^{\mathrm{Se}}$ and $\rho_{A}^{e}$. The distinction between these two mechanisms would require tomographic measurements, which is beyond the scope of this study.

*Rubidium segregation:* Anti-correlation of $\rho_{A}^{\mathrm{Rb}}$ and $I_{\mathrm{XBIC}}$ is unveiled in direct comparison of the corresponding maps in Figure 4b,c and in their negative correlation coefficient in Figure 6. This indicates that Rb accumulates at recombination-active defect sites (including grain boundaries), where the XBIC signal is weaker. This observation is in accordance with other studies, where Rb has been shown to segregate at grain boundaries [14,54,55].

In the case of the Rb distribution, lacking correlations are also relevant: $\rho_{A}^{\mathrm{Rb}}$ is not generally correlated with $\rho_{A}^{\mathrm{Se}}$ or $\rho_{A}^{e}$, which indicates that Rb does neither accumulate in voids, nor that it is homogeneously distributed in the absorber layer (which would be seen as negative and positive correlations, respectively). This lack of correlation is in accordance to previous findings [56]. Furthermore, it is noteworthy that the statistical distribution of Rb is similar in both groups in Figure 5.

*Performance:* The assessment of the nanoscale performance of a Cu(In,Ga)Se${}_{2}$ solar cell by two means—electrically by XBIC and optically by XEOL—conceptually allows the discrimination of measurement artifacts based on their different detection path of charge-carrier recombination: while XBIC measurements are affected by the electronic circuit of the entire solar cell, XEOL measurements are affected by the optical performance of the layer stack—a good solar cell is also a good light-emitting device due to the reciprocity principle [57].

Both performance measurements have in common that they depend, in first approximation linearly, on the X-ray absorptance of the absorber layer. Accordingly, the absorber topology has a strong impact on the XBIC and XEOL signal, which is indicated by the positive correlation between $\rho_{A}^{\mathrm{Se}}$, $\rho_{A}^{e}$, $I_{\mathrm{XBIC}}$, and $f_{\mathrm{XEOL}}$.

For the further improvement of solar cells, it is of particular interest to understand the origin of poor performance in areas that are not limited by topology but by non-radiative recombination, due to the presence of defects. Such areas can be seen in Figure 4a,c–e as an imperfect match of the maps and show up as different distributions of $\rho_{A}^{\mathrm{Se}}$, $\rho_{A}^{e}$, $I_{\mathrm{XBIC}}$, and $f_{\mathrm{XEOL}}$ in Figure 5.

## 4. Conclusions

We have demonstrated the feasibility of four-fold multi-modal operando measurements at a scanning X-ray microscopy beamline, and presented an approach to combine, for the first time, XRF, XBIC, XEOL, and ptychography, as proposed in Reference [58]. Recording simultaneously the signals from a Cu(In,Ga)Se${}_{2}$ solar cell has allowed for point-by-point correlation of the composition, electrical, and optical performance, as well as structure in a non-destructive way.

While the statistical significance of the present proof-of-principle study is limited, the multi-modal approach allows the direct correlation of nanoscale properties, leading to the identification of device-relevant defect mechanisms as demonstrated in two-fold multi-modal measurements already [12,59]. Four-fold multi-modal measurements, as we showcased here, will significantly reduce the ambiguity in interpretation and may unveil correlations that have not been detectable so far, thanks to the complementarity of XRF, XBIC, XEOL, and ptychography measurements.

In view of the diffraction-limited synchrotron facilities that will see light in the coming years, the presented development of sophisticated X-ray microscopy measurements is particularly promising: as the brilliance of the X-ray beam will be boosted by orders of magnitude [42,60,61,62], the flux of focused photons will increase, and the trade-off between high flux and high coherence that limits the spatial resolution and signal-to-noise ratio today will be relaxed or even avoided entirely.

## Data Availability

The data presented in this study are openly available in Figshare at DOI: 10.6084/m9.figshare.13517078.

## Supplementary Materials

The following are available online at https://www.mdpi.com/1996-1944/14/1/228/s1, Figure S1: Effective molar area density $\rho_{A}$ of Se, Cu, Ga, In, Rb and Zn extracted from scanning X-ray fluorescence measurements, Figure S2: (a) Relative phase shift, reconstructed with ptychography and (b) the relative area electron density calculated from that, Figure S3: Characteristic figures to determine the optimum group size *k* for *k*-means clustering from (a) the *elbow* and (b) *silhouette* method, Figure S4: Correlation coefficient of Se ($\rho_{A}^{\mathrm{Se}}$), In ($\rho_{A}^{\mathrm{In}}$), Ga ($\rho_{A}^{\mathrm{Ga}}$), Cu ($\rho_{A}^{\mathrm{Cu}}$), Rb ($\rho_{A}^{\mathrm{Rb}}$) and Zn ($\rho_{A}^{\mathrm{Zn}}$), of the X-ray beam-induced current ($I_{\mathrm{XBIC}}$), of the XEOL photon count rate ($f_{\mathrm{XEOL}}$) and of the relative electron area density ($\Delta \rho_{A}^{e}$) for the total data set as well as the two sub-sets with data from the *k*-means groups 1 and 2, respectively.

## Abbreviations

The following abbreviations are used in this manuscript:

CCD	Charged Coupled Device
FWHM	Full Width Half Maximum
SI	Supplementary Information
XBIC	X-ray Beam-Induced Current
XEOL	X-ray Excited Optical Luminescence
XRF	X-ray Fluorescence
