# Erratum: Ossig, C., et al. Four-Fold Multi-Modal X-ray Microscopy Measurements of a Cu(In, Ga)Se_2_ Solar Cell. *Materials* 2021, *14*, 228

**DOI:** 10.3390/ma14051189

**Published:** 2021-03-03

**Authors:** Christina Ossig, Christian Strelow, Jan Flügge, Andreas Kolditz, Jan Siebels, Jan Garrevoet, Kathryn Spiers, Martin Seyrich, Dennis Brückner, Niklas Pyrlik, Johannes Hagemann, Frank Seiboth, Andreas Schropp, Romain Carron, Gerald Falkenberg, Alf Mews, Christian G. Schroer, Tobias Kipp, Michael E. Stuckelberger

**Affiliations:** 1 Deutsches Elektronen-Synchrotron (DESY), Notkestr. 85, 22607 Hamburg, Germany; christina.ossig@desy.de (C.O.); jan.garrevoet@desy.de (J.G.); kathryn.spiers@desy.de (K.S.); martin.seyrich@desy.de (M.S.); dennis.brueckner@desy.de (D.B.); niklas.pyrlik@desy.de (N.P.); johannes.hagemann@desy.de (J.H.); frank.seiboth@desy.de (F.S.); andreas.schropp@desy.de (A.S.); gerald.falkenberg@desy.de (G.F.); christian.schroer@desy.de (C.G.S.); 2 Fachbereich Physik, Universität Hamburg, Luruper Chaussee 149, 22761 Hamburg, Germany; 3 Fachbereich Chemie, Universität Hamburg, Grindelallee 117, 20146 Hamburg, Germany; strelow@chemie.uni-hamburg.de (C.S.); jan.fluegge@chemie.uni-hamburg.de (J.F.); andreas.kolditz@chemie.uni-hamburg.de (A.K.); jan.siebels@chemie.uni-hamburg.de (J.S.); mews@chemie.uni-hamburg.de (A.M.); kipp@chemie.uni-hamburg.de (T.K.); 4 Empa, Überlandstrasse 129, 8600 Dübendorf, Switzerland; romain.carron@empa.ch

In the original version of our article [1], insufficient acknowledgement was given for providing and setting up the X-ray optics. We apologize for the original error. To correct this oversight, Frank Seiboth has been added as an author. The author contributions have been altered to more appropriately recognize the individual contributions.

The corrected author list and author contributions are provided below:

Corrected author list: Christina Ossig, Christian Strelow, Jan Flügge, Andreas Kolditz, Jan Siebels, Jan Garrevoet, Kathryn Spiers, Martin Seyrich, Dennis Brückner, Niklas Pyrlik, Johannes Hagemann, Frank Seiboth, Andreas Schropp, Romain Carron, Gerald Falkenberg, Alf Mews, Christian G. Schroer, Tobias Kipp, and Michael E. Stuckelberger.

Corrected author contributions: Conceptualization, M.E.S. and F.S.; project administration, M.E.S.; supervision, A.M., C.G.S., T.K., and M.E.S.; methodology, M.E.S.; resources, R.C., J.G., K.S., G.F., F.S., A.S., C.G.S., C.S., J.F., A.M., T.K., and M.E.S.; funding acquisition, A.M., G.F., and C.G.S.; investigation, C.O., C.S., J.F., A.K., J.S., D.B., J.H., F.S., A.S., T.K., and M.E.S.; formal analysis, C.O., C.S., M.S., N.P., F.S., A.S., R.C., and M.E.S.; visualization, C.O., M.S., N.P., A.S., and M.E.S.; writing—original draft, C.O. and M.E.S; writing—review and editing, C.O. and M.E.S. All authors have read and agreed to the published version of the manuscript.

The authors apologize for any inconvenience caused and state that the scientific conclusions are unaffected.
