# Supplementary material for: Four-Fold Multi-Modal X-ray Microscopy Measurements of a Cu(In,Ga)Se2 Solar Cell
Source: Materials (Basel). 2021 Jan 5;14(1):228. doi: 10.3390/ma14010228 (PMC7796438; doi:10.3390/ma14010228)
Supplement: Supplementary file 1 [file materials-14-00228-s001.pdf]

Supplementary Information

Four-Fold Multi-Modal X-ray Microscopy Measurements of a Cu(In,Ga)Se_2 Solar Cell

Christina Ossig[a,b], Christian Strelow[c], Jan Flügge[c], Andreas Kolditz[c], Jan Siebels[c], Jan Garrevoet[a], Kathryn Spiers[a], Martin Seyrich[a,b], Dennis Brückner[a,b], Niklas Pyrlik[a,b], Johannes Hagemann[a], Andreas Schropp[a], Romain Carron[d], Gerald Falkenberg[a], Alf Mews[c], Christian G. Schroer[a,b], Tobias Kipp[c], Michael E. Stuckelberger[a,*]

[a]*Deutsches Elektronen-Synchrotron (DESY), Notkestr. 85, 22607 Hamburg, Germany*
[b]*Universität Hamburg, Fachbereich Physik, Luruper Chaussee 149, 22761 Hamburg, Germany*
[c]*Universität Hamburg, Fachbereich Chemie, Grindelallee 117, 20146 Hamburg, Germany*
[d]*Empa, Überlandstrasse 129, 8600 Dübendorf, Switzerland*

S.1 X-Ray Fluorescence

The X-ray fluorescence data have been fitted with PyMca version 5.5.3 [1]. To correct for the background radiation, we subtracted an average background—gained from a fit of the spectra integrated over the scanned area—of each single spectrum and did not use further background stripping routines, as justified and detailed in [2]. To extract the mass fraction of the elements, we used PyMca's built-in routine, which is based on the fundamental parameter method [3, 4]. For the application of self-absorption correction, the nominal values for the sample structure and for the measurement geometry as described in sections 2.1 and 2.2 were considered as a priori information. We used Se as reference element to scale the mass fraction, as Se is the absorber element with highest signal-to-noise ratio and most homogeneous stoichiometric distribution. Based on the measured fluorescence count rate f_i and the unscaled mass fraction w_i^* obtained through PyMca for each element i, the effective molar area density ρ_A^i was calculated as follows.

First, the mass fraction was scaled for each element such that the sum equals 1 at each scanned spot:

$$w_i = \frac{w_i^*}{\sum_i w_i^*}. \tag{S.1}$$

By multiplying the scaled mass fraction w_i with the nominal thickness d_nom and mass density $\rho_\mathrm{nom}^\mathrm{mass,tot}$ of the layer, the nominal mass area density

$$\rho_\mathrm{A,nom}^{\mathrm{mass},i} = w_i^* \cdot d_\mathrm{nom} \cdot \rho_\mathrm{nom}^\mathrm{mass,tot} \tag{S.2}$$

*Corresponding author
Email address: `Michael.stuckelberger@desy.de` (Michael E. Stuckelberger)

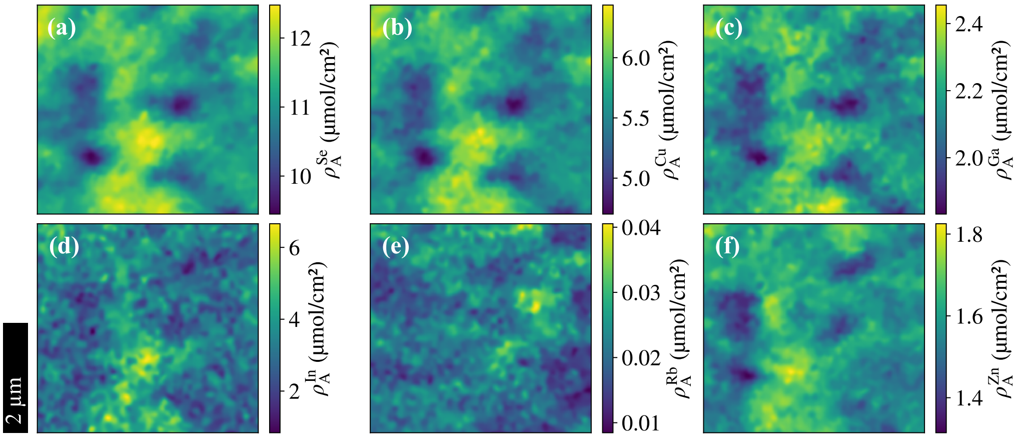

Figure S1: Effective molar area density ρ_A of Se, Cu, Ga, In, Rb and Zn extracted from scanning X-ray fluorescence measurements.

in [g/cm^2] was calculated for each element and converted into the nominal molar area density in [mol/cm^2] with the molar mass M_i:

$$\rho_{\text{A,nom}}^{\text{mol},i} = \frac{\rho_{\text{A,nom}}^{\text{mass},i}}{M_i}. \tag{S.3}$$

To obtain the effective molar area density map for each element i, the count rate f_i was scaled to $\rho_{\text{A,nom}}^{\text{mol},i}$ averaged over all scan points:

$$\rho_{\text{A}}^{i} = \frac{\overline{\rho_{\text{A,nom}}^{\text{mol},i}} \cdot f_i}{\overline{f_i}}. \tag{S.4}$$

For completeness, the resulting maps of all main absorber elements as well as Rb and Zn are shown in Figure S1.

S.2 X-ray Beam-Induced Current

To regain the measured current I_{XBIC} from the acquired count rate f^{XBIC}, the factors along the signal processing chain are considered in

$$I_{\text{XBIC}} = \frac{2 \cdot f^{\text{XBIC}} \cdot W_{\text{ff}}}{p \cdot k \cdot l}, \tag{S.5}$$

with $W_{\text{ff}} = \sqrt{2}$ taking into account the sine-shape of the modulated signal, $p = 10^6$ V/A the sensitivity of the pre-amplifier, $k = 10^6$ Hz/V from the voltage-to-frequency conversion, and $l = 10$ the scaling of the lock-in amplifier. The factor 2 takes into account the oscillation between the positive and negative amplitude [5].

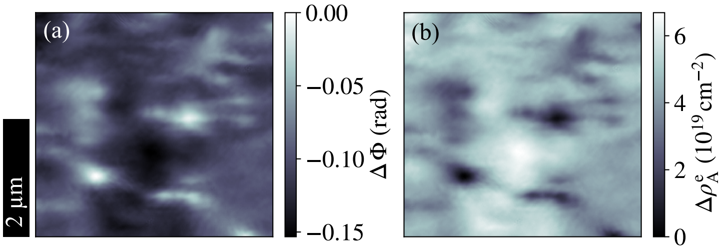

Figure S2: (a) Relative phase shift, reconstructed with ptychography and (b) the relative area electron density calculated from that.

S.3 X-ray Excited Optical Luminescence

As the interaction point between the sample and the X-ray beam was aligned to the focus spot of the confocal XEOL setup, the luminescence photons were projected to a reasonably narrow point on the CCD detector. For maximum signal-to-noise ratio, the photon count rate of the camera pixels were first integrated in the vertical direction of the detector and second fitted in horizontal direction by a Gaussian on top of a linear background.

S.4 Ptychography

The reconstruction of the phase shift Φ via ptychography was done with an in-house developed code [6]. Lacking of an absolute reference for the phase shift in this measurement, we have offset Φ such, that the minimal phase shift (maximal value of Φ) is zero; the resulting relative phase shift is denoted $\Delta\Phi$. The reconstructed image has a pixel size of 17 nm and is displayed in Fig. S2a.

The phase shift Φ is related to δ from Equation (2) through

$$\Phi = -\frac{2\pi}{\lambda}\int \delta(x)dx \tag{S.6}$$

where λ is the wavelength and x the direction of X-ray beam propagation [7]. With Equation (2) and the conversion of the electron density to the area electron density via

$$\rho_{\mathrm{A}}^{\mathrm{e}} = \int \rho_{\mathrm{e}}(x)dx, \tag{S.7}$$

the relative phase shift can be converted into the relative area electron density:

$$\Delta\rho_{\mathrm{A}}^{\mathrm{e}} = -\frac{\Delta\Phi}{\lambda \cdot r_{\mathrm{e}}}. \tag{S.8}$$

As introduced in Equation (2), r_{e} is the classical electron radius. Figure S2b shows the resulting relative area electron density map.

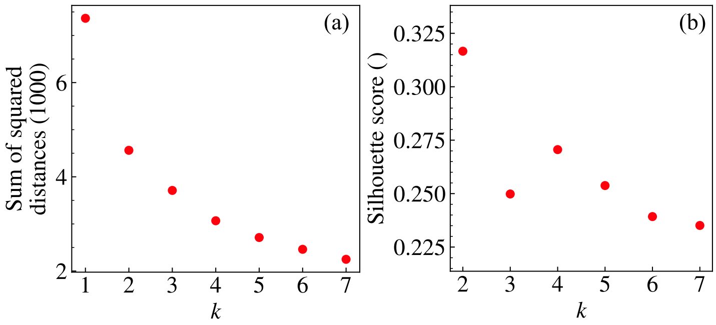

Figure S3: Characteristic figures to determine the optimum group size k for k-means clustering from (a) the *elbow* and (b) *silhouette* method.

S.5 Position Correction and Normalization

Within the ptychographic reconstruction, the position of the sample-beam interaction could be refined beyond the accuracy of the scanning system. Therefore, we have applied this position correction to the measurements from all modalities which required, however, the re-alignment of the reconstructed ptychographic image with the results obtained from the other modalities. The alignment was achieved through registration of the phase-shift image and the Se area density image using the enhanced correlation coefficient image alignment algorithm [8] from the OpenCV library [9]. All measurands except the ptychographic phase shift were interpolated on the supergrid given by the ptychographic reconstruction with the nearest neighbor method.

To compensate for the oversampling during the measurement, a Gaussian filter from the OpenCV library was applied. With a beam size of $105\,\mathrm{nm} \times 108\,\mathrm{nm}$ (FWHM), a filter size with a standard deviation of $\sigma = 54\,\mathrm{nm}$ was appropriate. For statistical correlation, the maps were cropped by half filter width on all sides to avoid artifacts from the filter at the border corrupting the correlation.

Furthermore, all measurements were normalized to the dwell time and incident photon flux.

S.6 k-Means Clustering

Given a set of n measurands spanning an n-dimensional space, the k-means clustering algorithm aims to separate the data into k groups of similar size and small variance by minimizing the sum of squared Euklidian distances between each data point and the center of the cluster it belongs to [10]. The k-means implementation of the scikit-learn library [11] in Python was employed in this study. The optimal number of groups k was determined using the so-called *elbow* and *silhouette* methods, both of which we implemented in Python using scikit-learn. The *elbow* refers to a kink in the plot of the minimized sum of squared Euklidian distances as a function of k. Details on the silhouette score can be found elsewhere [12]. In general, a silhouette score of 1 means that a perfect cluster structure has been found and a score of -1 translates to poor clustering. In Figure S3 it can be seen, that both methods indicate a group size of $k = 2$ to be ideal.

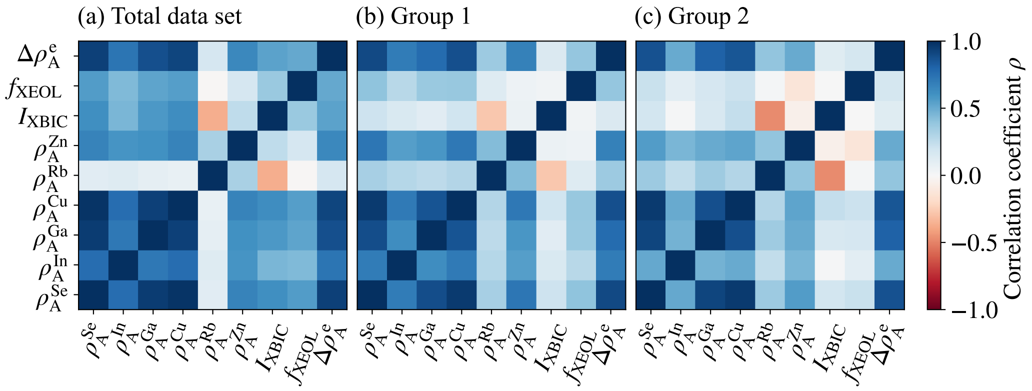

Figure S4: Correlation coefficient of Se (ρ_A^{Se}), In (ρ_A^{In}), Ga (ρ_A^{Ga}), Cu (ρ_A^{Cu}), Rb (ρ_A^{Rb}) and Zn (ρ_A^{Zn}), of the X-ray beam-induced current (I_{XBIC}), of the XEOL photon count rate (f_{XEOL}) and of the relative electron area density ($\Delta\rho_A^e$) for the total data set as well as the two sub-sets with data from the k-means groups 1 and 2, respectively.

S.7 Correlation Coefficients

The correlation coefficient ρ that is shown in the manuscript only for a subset of measurands is shown in Figure S4 for all measurands, with Figure S4a including the data from the entire maps and Figure S4b–c including only the data for the k-means groups 1 and 2, respectively.

References

[1] V. Solé, E. Papillon, M. Cotte, P. Walter, J. Susini, A multiplatform code for the analysis of energy-dispersive X-ray fluorescence spectra, Spectrochim. Acta Part B 62 (2007) 63–68.

[2] C. Ziska, Analysis of synchrotron-based x-ray fluorescence measurements from multiple detector modules, Bachelor's Thesis at Universität Hamburg (2020).

[3] D. K. G. de Boer, Calculation of x-ray fluorescence intensities from bulk and multilayer samples, X-ray Spectrometry 19 (1990) 145–154.

[4] A. Heginbotham, V. A. Solé, CHARMed PyMca, Part I: A Protocol for Improved Interlaboratory Reproducibility in the Quantitative ED-XRF Analysis of Copper Alloys, Archaeometry 49 (2017) 714–730. doi:10.1111/arcm.12282.

[5] C. Ossig, T. Nietzold, B. M. West, M. I. Bertoni, G. Falkenberg, C. G. Schroer, M. E. Stuckelberger, X-ray beam induced current measurements for multi-modal x-ray microscopy of solar cells, Journal of Visual Experiments (2019) e60001doi:10.3791/60001.

[6] A. Schropp, R. Hoppe, J. Patommel, D. Samberg, F. Seiboth, S. Stephan, G. Wellenreuther, G. Falkenberg, C. G. Schroer, Hard x-ray scanning microscopy with coherent radiation: Beyond the resolution of conventional x-ray microscopes, Applied Physics Letters 100 (25) (2012) 253112. arXiv:https://doi.org/10.1063/1.4729942, doi:10.1063/1.4729942.

[7] A. Diaz, P. Trtik, M. Guizar-Sicairos, A. Menzel, P. Thibault, O. Bunk, Quantitative x-ray phase nanotomography, Physical Review B 85 (2012) 020104(R). doi:10.1103/PhysRevB.85.020104.

[8] G. D. Evangelidis, E. Z. Psarakis, Parametric image alignment using enhanced correlation coefficient maximization, IEEE Computer Society 30 (2008) 1–8.

[9] OpenCV, Open source computer vision library, available at: https://opencv.org, (accessed June 19, 2020) (2015).

[10] T. Hastie, R. Tibshirani, J. Friedman, The Elements of Statistical Learning: Data Mining, Inference, and Prediction, 2nd Edition, Springer-Verlag New York, 2009. doi:10.1007/978-0-387-84858-7.

[11] F. Pedregosa, G. Varoquaux, A. Gramfort, V. Michel, B. Thirion, O. Grisel, M. Blondel, P. Prettenhofer, R. Weiss, V. Dubourg, J. Vanderplas, A. Passos, D. Cournapeau, M. Brucher, M. Perrot, E. Duchesnay, Scikit-learn: Machine learning in Python, Journal of Machine Learning Research 12 (2011) 2825–2830.

[12] P. J. Rousseeuw, Silhouettes: a graphical aid to the interpretation and validation of cluster analysis, Journal of computational and applied mathematics 20 (1987) 53–65.
